# Supplementary material for: Osteosarcoma-Derived Extracellular Vesicles Induce Lung Fibroblast Reprogramming
Source: Int J Mol Sci. 2020 Jul 30;21(15):5451. doi: 10.3390/ijms21155451 (PMC7432951; doi:10.3390/ijms21155451)
Supplement: Supplementary file 1 [file ijms-21-05451-s001.zip › ijms-878431supplementary/Supplementary Figures and Figure Legends - Revision.docx]

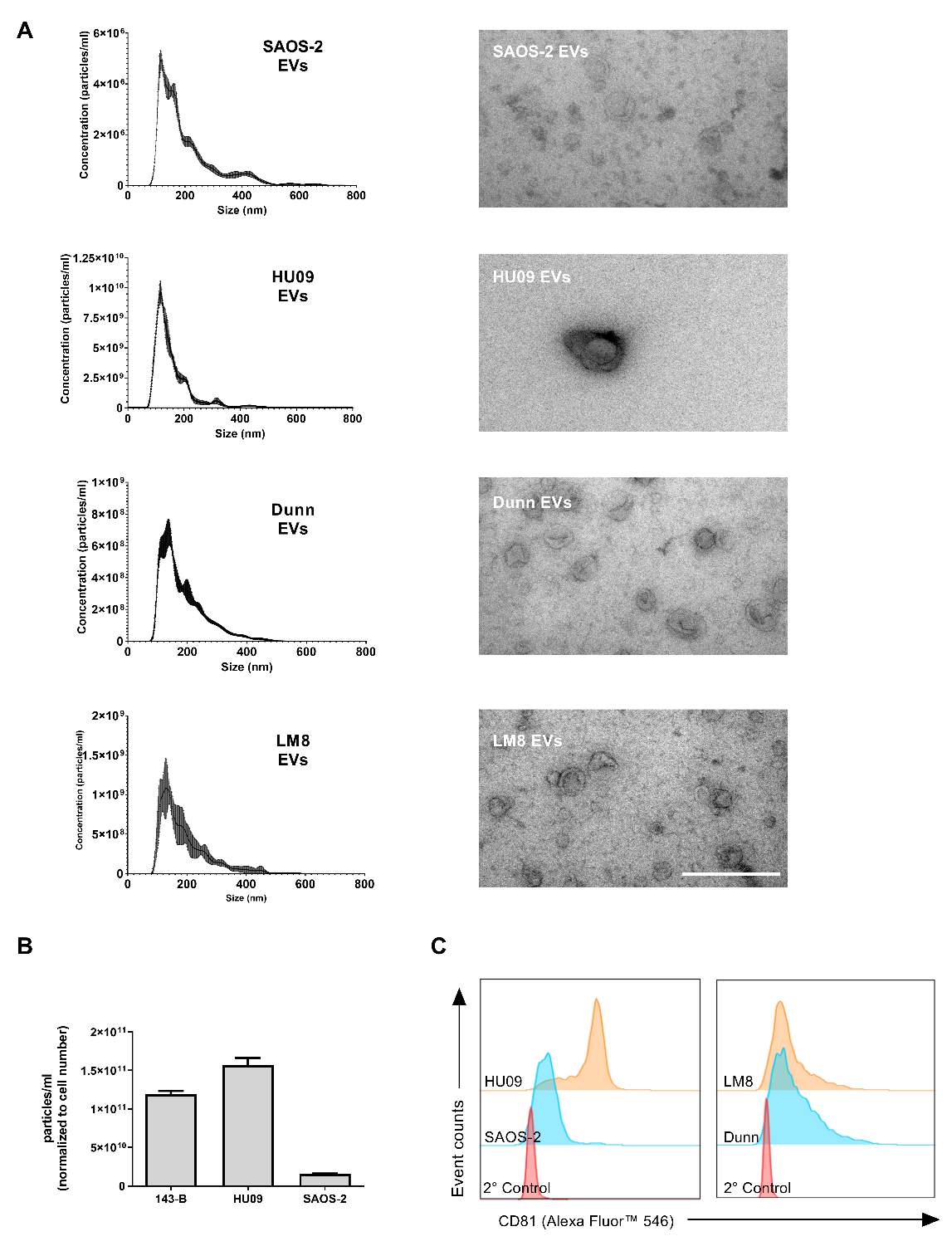
**Supplementary Figures and Figure Legends**

**Figure S1.** Characterization of human and murine osteosarcoma-derived EVs. (**A**) Concentration and size distribution assessed by Nanosight analysis of indicated (left panels) cell line-derived EVs and respective representative electron micrographs (right panels). Scale bar, 500nm; (**B**) Production rate of EVs for indicated cell lines per 1,000,000 cells; (**C**) Representative histograms of CD81 flow cytometry analysis of beads coated with EVs derived from indicated osteosarcoma cell lines.


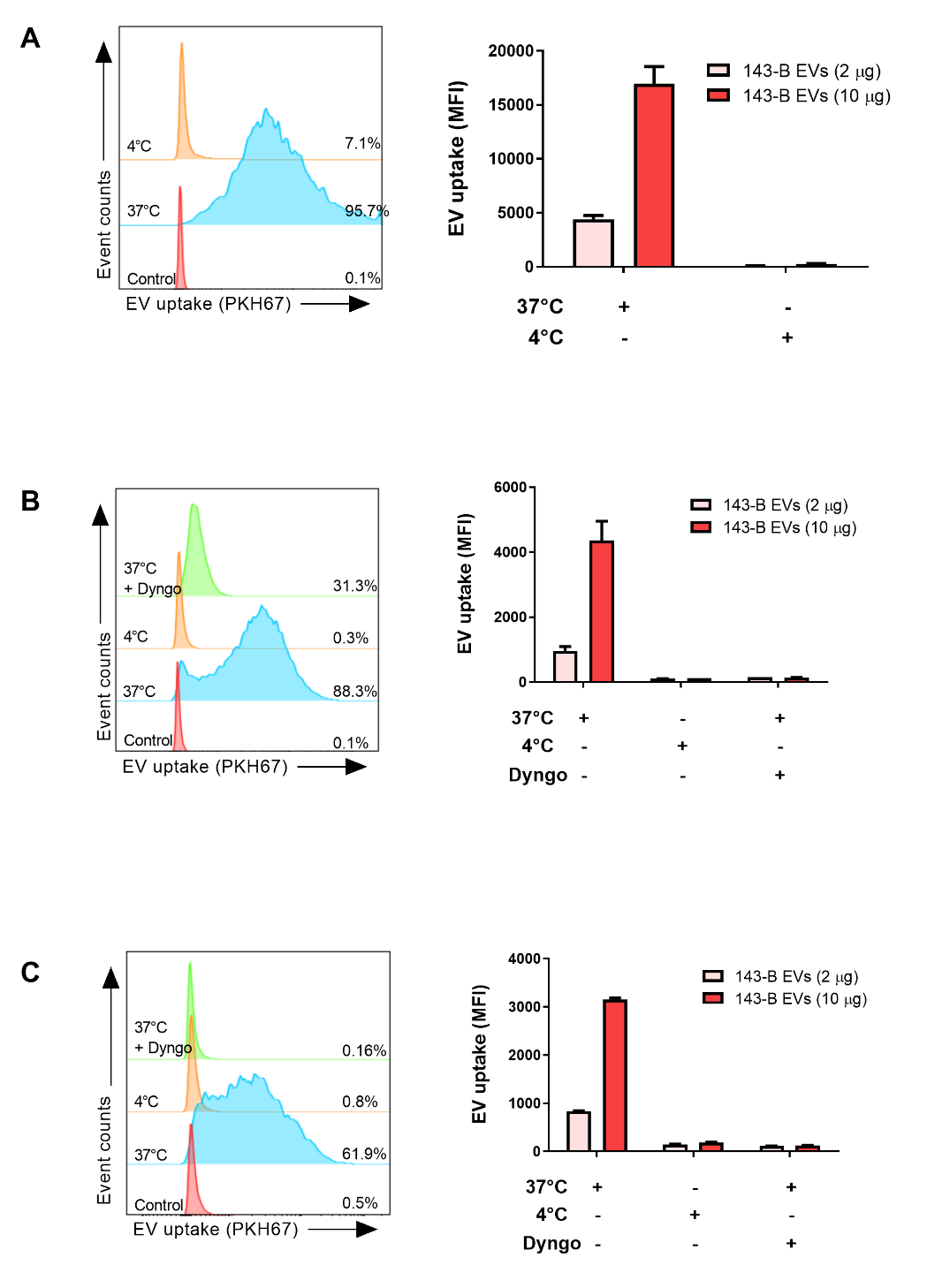


**Figure S2.** 143-B EV internalization by recipient cells is an active, energy-dependent endocytic process. Uptake of PKH67labelled 143-B EVs by HUVEC (**A**), WI38 (**B**) and MRC-5 (**C**) cells was assessed after 4 hours long incubation either at 37°C or 4°C in the presence/absence of Dyngo-4a (5 µM). Internalization was measured by flow cytometry. Representative histograms indicating the percentage of cells internalizing EVs (10 µg) (left panels) and quantification of mean fluorescence intensity (MFI) (right panels). Data represent means ± SEM from three independent experiments.


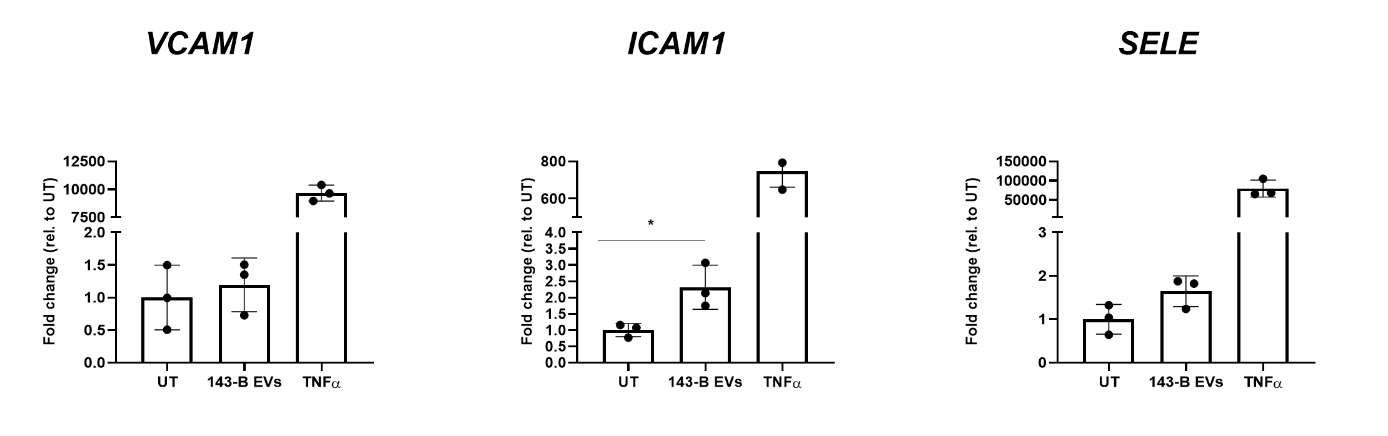


**Figure S3.** Effects of 143-B EVs on mRNA levels of adhesion molecules in HUVEC cells. Relative mRNA expression levels of indicated genes assessed by RT-PCR in HUVEC cells after incubation with 143-B EVs (50 µg/ml) for 6 hours. Transcripts were normalized to *GAPDH* and expressed as fold change relative to the untreated control (UT). Three independent experiments were performed. * *p* < 0.05, unpaired Student's t test.


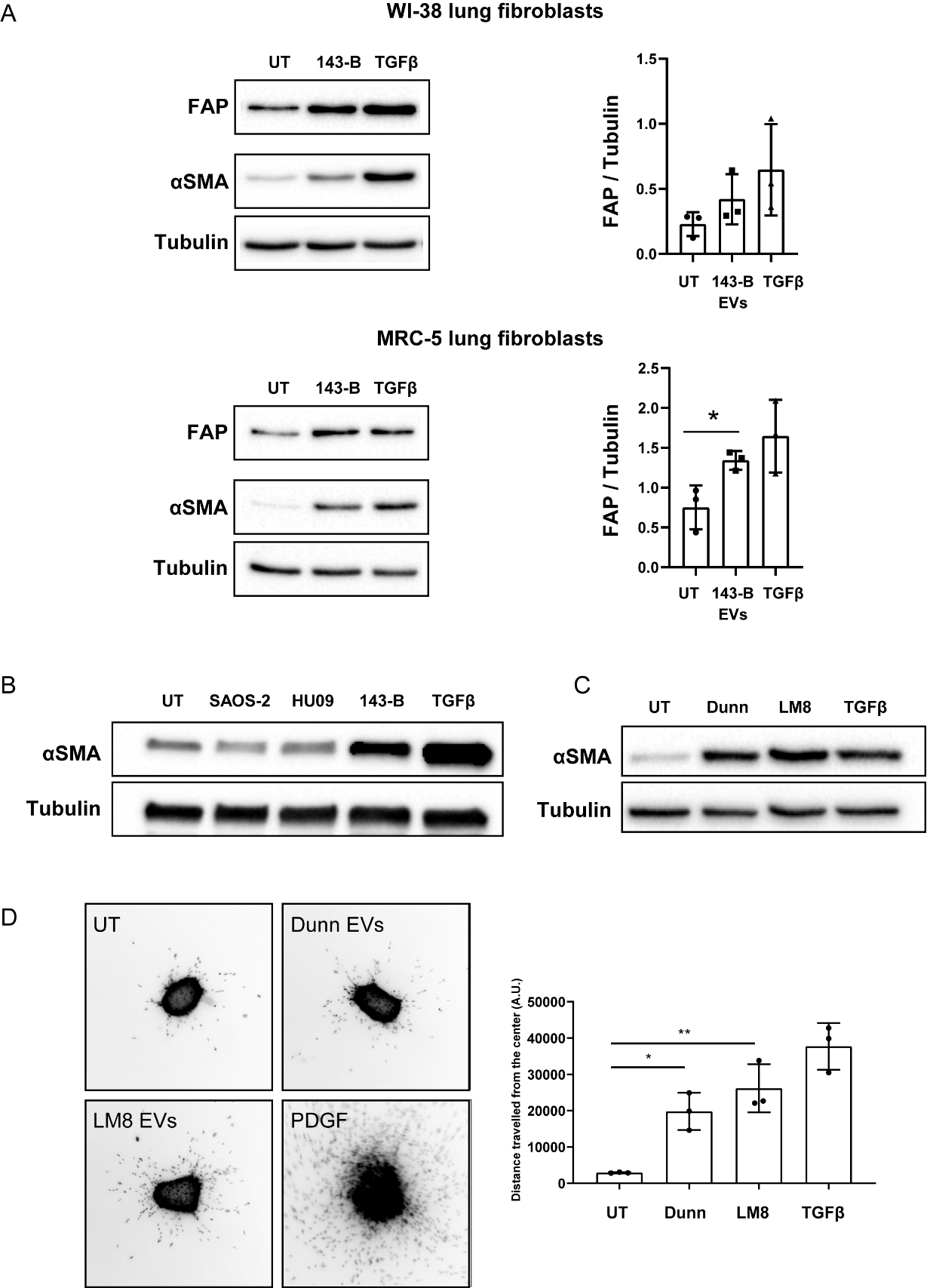


**Figure S4.** Effects of human and murine osteosarcoma -derived EVs on lung fibroblast differentiation in vitro. (**A**) FAP and αSMA protein levels in whole cell extracts of lung fibroblasts treated with 143-B-derived EVs (20 μg/ml) or soluble TGFβ1 for 48 hours were examined by Western blot analysis. Tubulin was used as a protein loading control. Representative Western blots (left panels) and respective quantitative analysis of three independent experiments (right panels). (**B**) Western blot analysis of αSMA protein levels in whole cell extracts of WI-38 lung fibroblasts treated with either a panel of human osteosarcoma cell line-derived EVs (20 µg/ml) or murine osteosarcoma -derived EVs (20 µg/ml) and soluble TGFβ1 (10 ng/ml) for 48 hours. Tubulin was used as the protein loading control. Representative Western blots of two independent experiments; (**C**) Representative images of the spheroid invasion assay in MRC-5 fibroblasts treated with Dunn EVs (20 μg/ml) or LM8 EVs (20 μg/ml) or PDGF (20 ng/ml) for 48 hours (left panels) and quantification of distances from spheroids centers using cell dissemination counter software aSDIcs (right panels). (* *p* < 0.05, ** *p* < 0.01, Bonferonni's one-way ANOVA test).


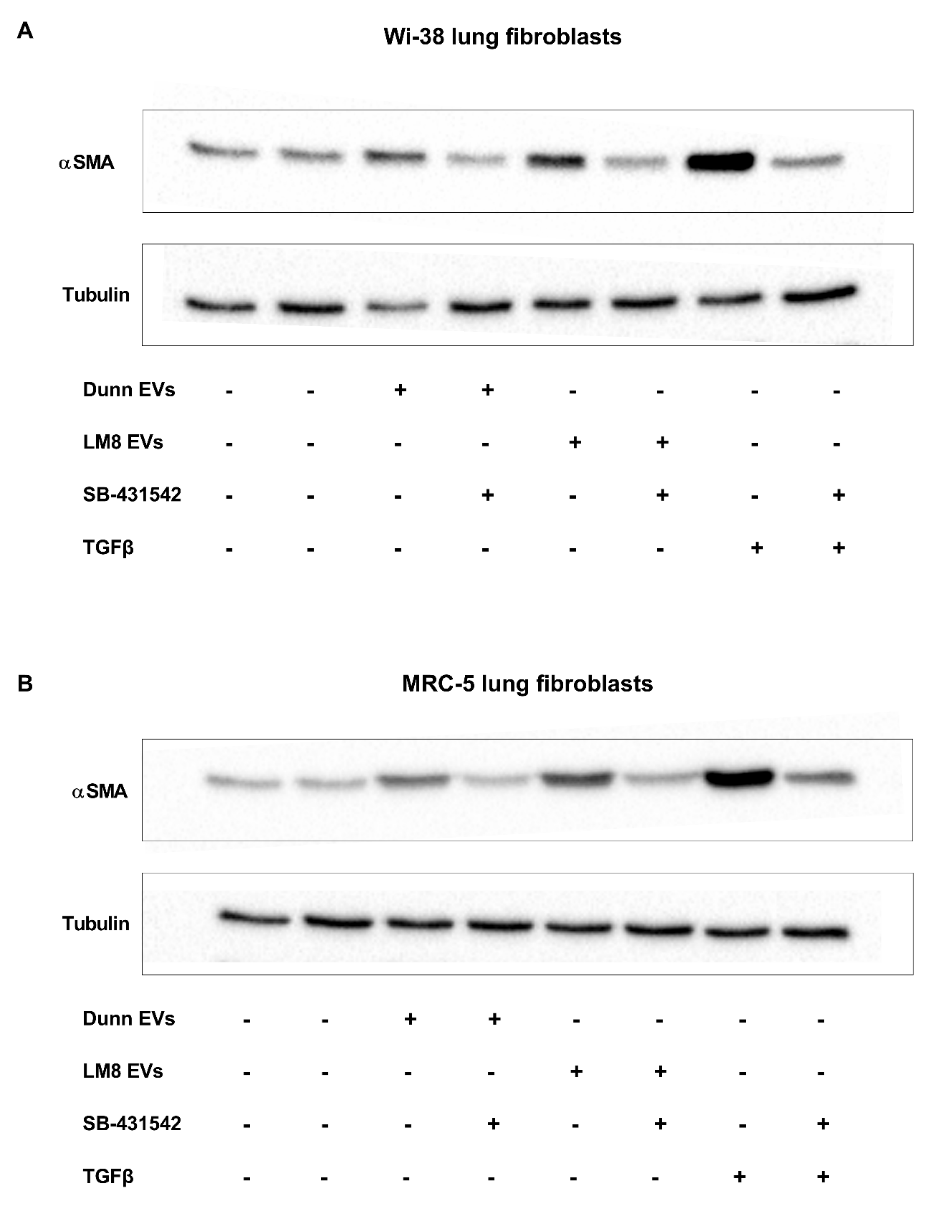


**Figure S5.** Murine osteosarcoma cell-derived EVs trigger lung fibroblast differentiation through TGFβ in vitro. Western blot analysis of αSMA protein expression in WI-38 (**A**) and MRC-5 (**B**) fibroblasts upon exposure to Dunn- or LM8-derived EVs (20 µg/ml) or soluble TGFβ1 (10 ng/ml) for 48 hours with or without pre-treatment with TGFβ receptor 1 inhibitor SB-431542 (10 μM) for 30 minutes. Representative Western blots of two independent experiments


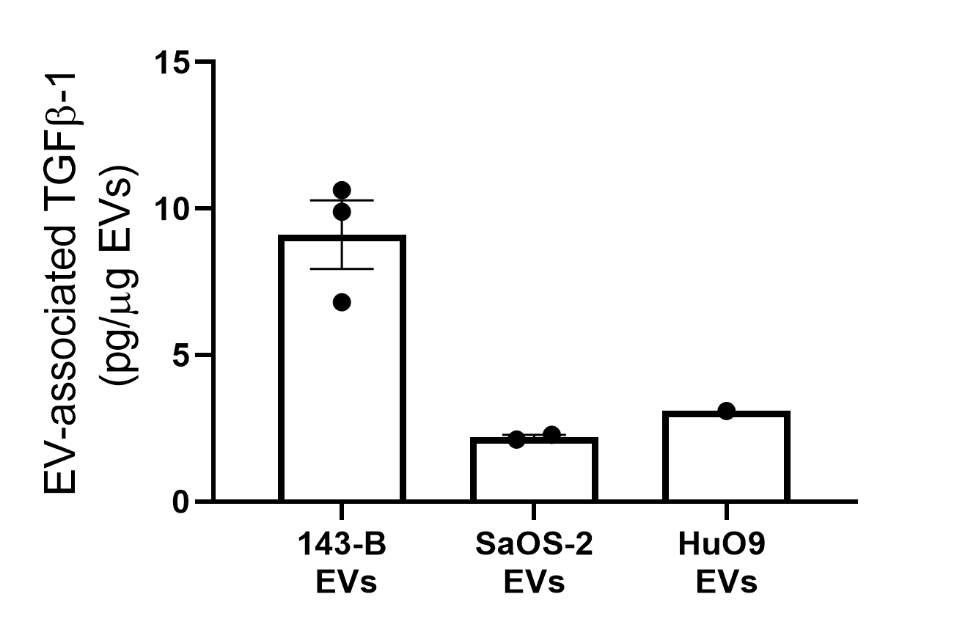


**Figure S6.** EV-associated TGFβ is higher in 143-B EVs than SAOS-2 and HU09 EVs. 10 µg of EVs from independently isolated extracts were activated and used for ELISA analysis.
