# Supplementary material for: Osteosarcoma-Derived Extracellular Vesicles Induce Lung Fibroblast Reprogramming
Source: Int J Mol Sci. 2020 Jul 30;21(15):5451. doi: 10.3390/ijms21155451 (PMC7432951; doi:10.3390/ijms21155451)
Supplement: Supplementary file 1 [file ijms-21-05451-s001.zip › ijms-878431supplementary/Supplementary Tables.docx]

**Supplementary Table S1.** List of primers utilized for Real-Time PCR.

| **Gene Name** | **Forward Primer**  **Sequence (5'-3')** | **Reverse Primer**  **Sequence (5'-3')** |
| --- | --- | --- |
| *IL6* | AGA GCT GTG CAG ATG AG | GCT GGC ATT TGT GGT TG |
| *TGFB1* | GCC TTT CCT GCT TCT CAT | CGT GGA GCT GAA GCA AT |
| *TGFB2* | CTT CCC CTC CGA AAC TGT CTG | ATC AAG GTA CCC ACA GAG CAC |
| *TGFB3* | CCT CCA AGG TTT TCC GCT TC | CAT CTG GCC GAA GGA TCT GG |
| *ACTA2* | TCA ATG TCC CAG CCA TGT AT | CAG CAC GAT GCC AGT TGT |
| *FN1* | AGG AAG ATG TTG CAG AGT T | AGA CAC TGG AGA CAC TCA CTA |
| *VEGFA* | CAT CTT CAA GCC ATC CTG T | TGA GGT TTG ATC CGC ATA AT |

**Supplementary** **Table S2.** Guide RNAs for CRISPR-Cas9 -mediated deletion of TGFβ-1

| **Name** | **Sequence (5'-3')** |
| --- | --- |
| *gRNA_KO#1_TGFb-1_1078-1100* | CAC CGT GTA CAA CAG CAC CCG CGA C |
| *gRNA_KO#2_TGFb-1_1404-1426* | CAC CGA ACC ACT CTG GCG AGT CGC T |
| *gRNA_human_non-targeting control* | GAC TTA TAA ACT CGC GCG GA |
